# Tempo-Spatial Pattern of Stepharine Accumulation in *Stephania Glabra* Morphogenic Tissues

**DOI:** 10.3390/ijms20040808

**Published:** 2019-02-13

**Authors:** Tatiana Y. Gorpenchenko, Valeria P. Grigorchuk, Dmitry V. Bulgakov, Galina K. Tchernoded, Victor P. Bulgakov

**Affiliations:** 1 Federal Scientific Center of the East Asia Terrestrial Biodiversity (Institute of Biology and Soil Science), Far Eastern Branch of the Russian Academy of Sciences, 159 Stoletija Str., 690022 Vladivostok, Russia; kera1313@mail.ru (V.P.G.); bulgakov-dv@mail.ru (D.V.B.); galcher45@rambler.ru (G.K.T.); 2 Far Eastern Federal University, School of Biomedicine, 8 Sukhanova Str., 690950 Vladivostok, Russia

**Keywords:** alkaloids localization, plant cell culture, stepharine, biotechnology, MALDI-MS, Menispermaceae

## Abstract

Alkaloids attract great attention due to their valuable therapeutic properties. Stepharine, an aporphine alkaloid of *Stephania glabra* plants, exhibits anti-aging, anti-hypertensive, and anti-viral effects. The distribution of aporphine alkaloids in cell cultures, as well as whole plants is unknown, which hampers the development of bioengineering strategies toward enhancing their production. The spatial distribution of stepharine in cell culture models, plantlets, and mature micropropagated plants was investigated at the cellular and organ levels. Stepharine biosynthesis was found to be highly spatially and temporally regulated during plant development. We proposed that self-intoxication is the most likely reason for the failure of the induction of alkaloid biosynthesis in cell cultures. During somatic embryo development, the toxic load of alkaloids inside the cells increased. Only specialized cell sites such as vascular tissues with companion cells (VT cells), laticifers, and parenchymal cells with inclusions (PI cells) can tolerate the accumulation of alkaloids, and thus circumvent this restriction. *S. glabra* plants have adapted to toxic pressure by forming an additional transport secretory (laticifer) system and depository PI cells. Postembryonic growth restricts specialized cell site formation during organ development. Future bioengineering strategies should include cultures enriched in the specific cells identified in this study.

## 1. Introduction

Alkaloid-producing plants have developed effective coping strategies inherent to secondary metabolism, including competition for resources, metabolic imbalance, and potential self-intoxication [1]. Efforts to improve the yield of alkaloids from cell cultures have focused on feeding precursors or over-expressing the transcription factors and enzymes positioned at metabolic bottlenecks; however, they have largely only resulted in modest improvements in yield [2,3,4]. Effective metabolic engineering approaches are often obscured by limitations in our knowledge of alkaloid biosynthesis regulation. Progress in systems biology will likely greatly impact the biotechnological production of alkaloids [5]. However, systems biology approaches in terms of secondary metabolism are developing slowly, especially for proteomics and bioinformatics studies of the relationship between protein regulation of secondary metabolism and the modules that determine the stem cell specification, embryogenesis, plant development, and tissue differentiation in general. In this respect, investigation into the general rules and specificity of the spatial-temporal regulation of biosynthetic pathways is of high importance.

Aporphine alkaloids belong to a large group of isoquinoline alkaloids [6,7]. These alkaloids possess considerable biological activity and are widely used in traditional western and eastern medicine, like many other secondary metabolites [3,8,9]. Stepharine is one of these aporphine alkaloids, exhibiting anti-aging, anti-hypertensive, and anti-viral effects [10,11,12]. Although some attempts have been made to induce appreciable stepharine biosynthesis in cultured *Stephania glabra* cells, success has been limited. Isoquinoline alkaloid biosynthesis has been examined for some economically relevant alkaloids, for example, morphine, sanguinarine and noscapine [13,14,15,16,17], MetaCyc Pathway. Despite these substances having similar precursor pools and metabolic pathways (S-reticuline/protoberberine pathway) as stepharine, the biosynthesis of aporphine alkaloids is yet to be investigated in detail. As described in our previous examination [18], *S. glabra* is the species that did not form calli on agar media. Explants of different plant portions (stem, leaf, root and tuber) did not yield calli, and calli were induced using young leaf explants immediately transferred into liquid media [18].

The cellular localization of alkaloid biosynthesis is diverse and complex [15,19]. A majority of plant alkaloids accumulate in laticifer cells (specialized internal secretory cells) and vascular bands have been shown to contain alkaloid biosynthetic enzymes. The identification of the relationship between the location and function of alkaloids represents a great challenge primarily due to metabolite transport between compartments and the complex metabolic network that exists in both time and space [20]. Furthermore, different developmental programs may regulate the biosynthesis of alkaloids [3,21]. In the opium poppy (*Papaver somniferum*), the differential expression of tyrosine decarboxylase genes, which catalyzes the formation of tyramine and dopamine and represents the first step in the biosynthesis of tetrahydroisoquinoline alkaloids, is restricted to the metaphloem and the protoxylem in the vascular bundles of the mature aerial organs of these plants [22]. Data presented by Onoyovwe et al. [22] showed that the central pathway from (S)—norcoclaurine to (S)—reticuline occurs exclusively in sieve elements. The early morphinan branch pathway enzymes have been found in sieve elements, but can also occur in laticifers and the final three enzymes involved in the conversion of thebaine to morphine are abundant in both areas. Meadow rue (*Thalictrum thalictroides*) does not contain laticifers and accumulates protoberberine alkaloids in its endodermal cells, which are distributed throughout the pith and cortex in rhizomes [23]. As in whole plants, laticifer-like cells can also be formed and cultured in vitro [24,25,26,27].

Advancing technologies based on a combination of microdissection, matrix-assisted laser desorption/ionization mass spectrometry (MALDI-MS) and tandem mass spectrometry (MS/MS) or MALDI-imaging, have been successfully used to study the localization of secondary metabolites in plants. The imaging of the tissue/cell-specific localization of secondary metabolites contributes to our understandings of their function and illuminates the major nodes of biosynthesis and transportation [28,29,30,31,32]. For example, the determination of alkaloids within *Sinomenium acutum* isolated through laser microdissection revealed their differential distribution in stems [33]. Sinomenine levels were highest in the phloem, while magnoflorine was highest in the outer portion of the cortex and sinoacutine accumulated in the xylem. The authors detected peaks of stepharine by accurate mass-spectrometry data, but could not pinpoint the metabolite’s distribution because of its small quantities [33]. Zeng et al. [34] used transcriptomic, proteomic, and metabolomic data to show that in Macleaya spp., alkaloids derived from (S)—reticuline (sanguinarine, chelerythrine, protopine, allocryptopine) accumulated in different organs, and roots were the predominant site of biosynthesis.

In the present study, we examined the accumulation of stepharine at the cellular level in cultured *S. glabra* tissues and mature plants. We identified specialized sites for stepharine accumulation and showed that the presence of specialized cells is critical for stepharine production.

## 2. Results

### 2.1. Generation of S. glabra Cell Lines

**Figure 1:**
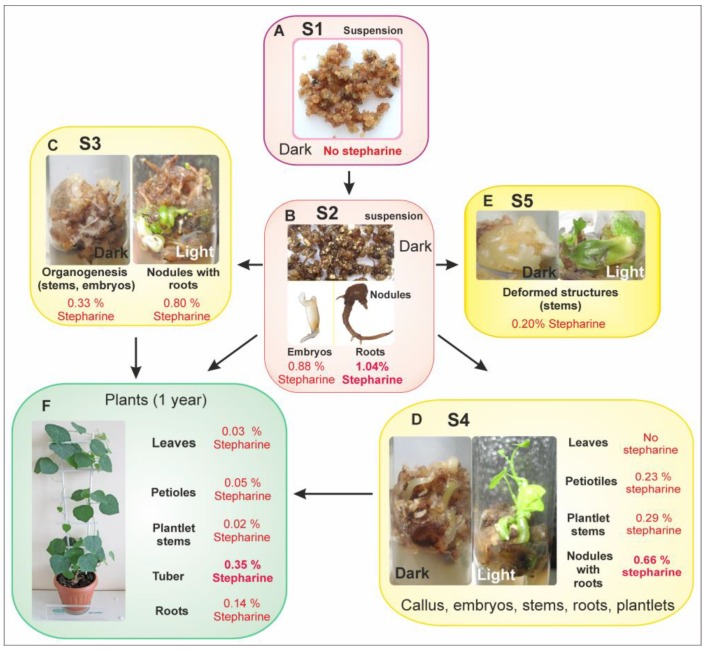
Callus lines and stepharine content in different organs and structures of *S. glabra*. (**A**) Proembryogenic S1 line. (**B**) Morphogenic S2 line. (**C**) Organogenic S3 line. (**D**) Organogenic S4 line. (**E**) Cell line S5 with deformed structures. (**F**) One-year-old plant.

The initial suspension-cultivated calli (S1) were used to obtain a generation of morphogenic cell cultures. Selection of small cell aggregates from this line yielded several suspension and callus lines (S2, S3, S4 and S5). In the morphogenic cell lines, unorganized aggregates of calli with roots, somatic embryos (SEs) at different stages of development, buds, and stems were obtained. Torpedo embryos of the two cell lines (S2, S3) reached the cotyledonary stage and were transferred into hormone-free media, rooted, and converted to plants with healthy phenotypes (Figure 1F). The S2, S3, S4, and S5 cell lines maintained their stable morphogenetic potential and high stepharine production over three years of cultivation.

### 2.2. Morphological and Histological Analysis of Cell Lines

**S1** The original callus tissue presented as slow-growing, uniform, small spherical aggregates approximately 2–4 mm in diameter (Figure 1A). After several subcultures, the S1 suspension culture formed small clusters consisting of densely stained meristematic cells (83–91% of total cells) and parenchymal cells. Large vacuolated parenchymal cells occupied the center of the bigger aggregates. The degradation of the old tissue was observed between the inner layers of the parenchymal cells and the upper layers of the meristematic cells in large aggregates. Then, these large aggregates scattered into smaller portions and the processes of callus formation started again. The presence of dark-colored metaphase nuclei and two and more nucleoli per nucleus indicated high cell division activity. No structural or tissue differentiation in the S1 cell line was observed over time.

**Figure 2:**
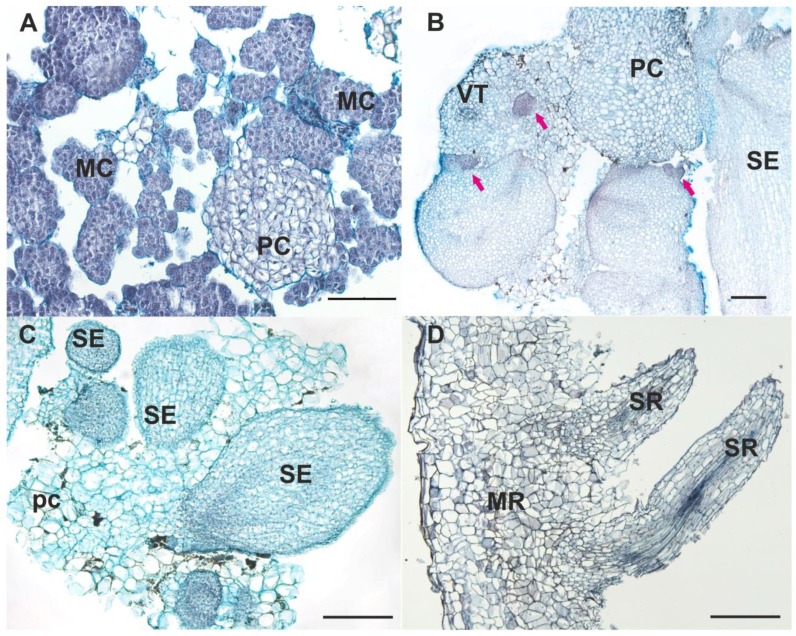
Histology of the *S. glabra* cell lines. (**A**) Parental proembryogenic cell line S1. (**B**) Morphogenic cell line S2 with SEs and initial meristematic centers (pink arrows). (**C**) Different stages of SEs in the S2 cell line. (**D**) Main root with secondary roots in the S2 cell line. MC, meristematic cells; PC, parenchymal cell; SE, somatic embryo; VT, vascular tissue; MR, main root; SR, secondary root. Bars, 200 µM.

**Figure 3:**
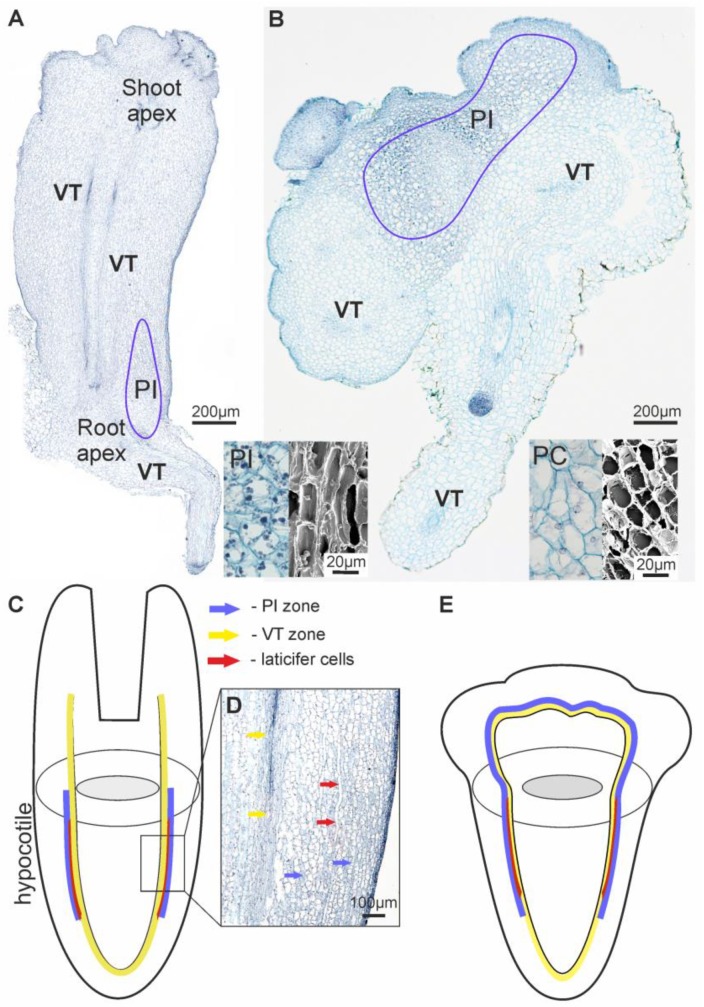
Histological images and schematic representations of the S2 differentiated structures. (**A**) A somatic embryo at the last torpedo stage. (**B**) A nodule with root. (**C**) A schematic representation of stepharine localization in a somatic embryo. (**D**) Histology section of the hypocotyl portion of a somatic embryo. (**E**) A schematic representation of stepharine localization in a nodule with root. Blue arrow and lines indicate localization of PI cells; red arrow indicates laticifer cells; yellow arrow and lines indicate VT cells. PC, parenchymal cells (histology and scanning electron microscopy (SEM) view); VT, vascular tissue with companion cells; PI, parenchymal cells with inclusions (histology and SEM view).

**S2** The cell suspension produced structures consisting of a mix of roots, nodules with roots, and SEs at different stages of development up to torpedo (Figure 1B). Histological investigation showed secondary indirect somatic embryogenesis (Figure 2B,C). The number of friable undifferentiated meristematic calli in the S2 line decreased drastically in comparison with the initial S1 line. Histological analysis demonstrated that the SEs and roots were produced by the simultaneous division of several cells and thus have a multicellular origin. Further somatic embryogenesis proceeded asynchronously through recognizable developmental stages including the globular, heart, and torpedo stages (Figure 1B, Figure 2B,C and Figure 3A). SEs were well-shaped and bipolar, with poorly developed cotyledonary leaves. Histology sections displayed clear tissue organization inside the SEs. Histological analysis of serial sections revealed that the structure of the elongated cells at the hypocotyls of the SEs resembled articulated anastomosing laticifer cells (Figure 3D). Occasionally, anomalous SEs were also observed.

In addition to SEs, nodules with roots were also observed (Figure 1B and Figure 3B). This indicates that cells of the calli could acquire diverse specialization patterns of competence. Roots were brown and well-formed (Figure 1B). The origin of the roots could be traced back to the inner tissue of the primary root itself when it was still within the upper portion of the parent root. The upper region of the nodules with root consisted of rounded clusters with two or three layers of small epidermal cells and a number of layers formed by large PI cells (Figure 3B). Histological sections of the roots also revealed well-differentiated VT cells (Figure 2D and Figure 3A,B).

The cultivation conditions did not allow the morphogenic structures of *S. glabra* calli to reach the final stages of development, but instead switched to additional secondary embryogenesis. During cultivation, many SEs stopped developing, overgrew and gave rise to new rounds of SEs, roots, or nodules with root. For further investigation into stepharine biosynthesis, meristematic structures, and aggregates of the S2 line were transferred to solid media with plant hormones as indicated below.

Scanning electron microscopy (SEM) revealed rounded grains and crystals in the companion cells of the VT, laticifers, PI cells in SEs, and nodules with root at different stages of development (Figure 3A,B). These grains and crystals were not observed in the PC cells. 

**S3** After transferring cell aggregates from the S2 culture onto solid media, containing 1.0 mg L^−1^ NAA the morphogenic potential of the culture changed. The meristematic center often produced deformed structures. The frequency of somatic embryogenesis was low and the development of SEs was blocked. The upper apexes of the SEs developed into weak stems. In light conditions, only the upper portion of the SEs became green. The number of friable unorganized PCs increased. The roots were light brown and became downy and resembled adventive secondary roots (Figure 1C). 

**S4** S4 calli were cultivated on solid media with 0.5 mg L^−1^ BA and 1.0 mg L^−1^ NAA (Figure 1D). Under these cultivation conditions, organogenesis was more common than embryogenesis. Nevertheless, occasional secondary SEs formed and with healthy morphologies, a main root, hypocotyls, and small cotyledons. The exposure of these SEs to light resulted in color changing and the development of the upper apex into a stem with leaves (Figure 1F). Longer cultivation allowed for the separate development of either shoots or roots. The histology sections demonstrated clusters of densely stained cells producing meristematic domes that formed primordia. Subsequently, these young adventitious primordial domes developed into stems. In some cases, the meristematic domes formed a root apex that developed into big roots similar to those in the S3 line. The S3 and S4 lines produced similar numbers of undifferentiated calli.

**S5** A higher concentration of BA (1.25 mg L^−1^) negatively affected the culture. No SEs or roots formed. Differentiated structures turned rigid and solid (Figure 1E). Under light cultivation, the callus structure did not change but the calli became green.

### 2.3. Analysis of Stepharine Content in Morphogenic Cell Lines of S. glabra

**Figure 4:**
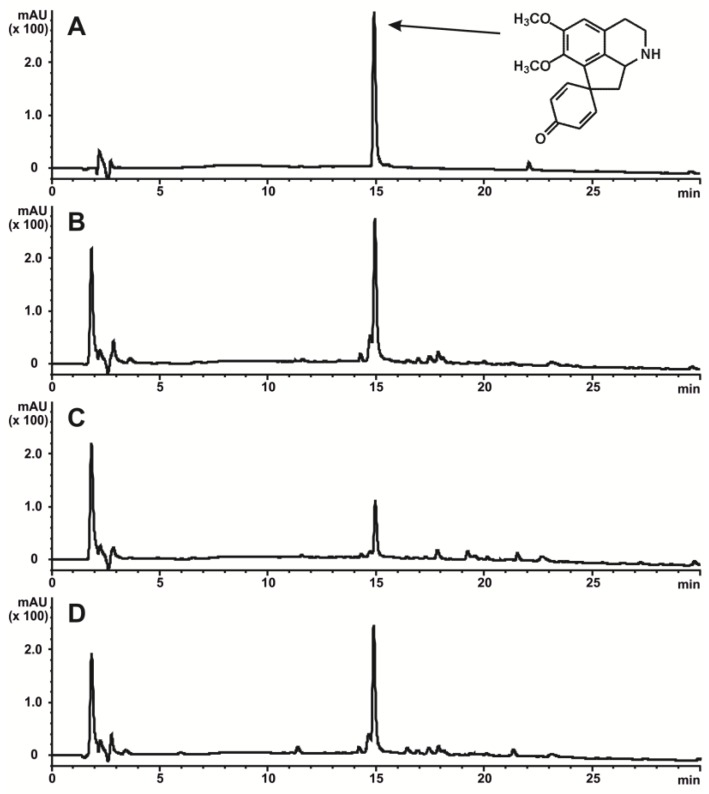
A representative HPLC-UV profile of the extracts obtained from cells of *S. glabra* (recorded at 237 nm). (**A**) The profile of the standard solution of stepharine and its molecular structure. (**B**) The extract from the nodules with roots of the S2 culture. (**C**) The extract from the nodules with roots of the S3 culture. (**D**) The extract from the nodules with roots of the S4 culture.

In our previous experiments, stepharine in *S. glabra* cultures was identified using the high-resolution tandem mass-spectrometry and their structure was confirmed by ^1^H and ^13^C NMR [17]. In the present work, the chromatographic conditions for stepharine analysis were optimized to obtain better separation efficiency of the alkaloids. The stepharine content in different tissues is summarized in Table 1. The accumulation of stepharine in cell lines at different stages of organ development is presented in Table 1. The chromatographic profiles were similar for all cell lines, indicating the uniformity of alkaloid patterns (Figure 4). No stepharine was detected in the initial cell culture S1 or the young leaves of the plantlets. The greatest amount of stepharine was found in the nodules with roots of the S2 line, 1.04 ± 0.03% dry weight (DW). Similar levels of stepharine were found in the nodules with roots of the S3 and S4 lines, 0.80 ± 0.19% and 0.66 ± 0.06% DW respectively (Table 1).

### 2.4. Distribution of Stepharine Determined by MALDI-MS Analysis

**Figure 5:**
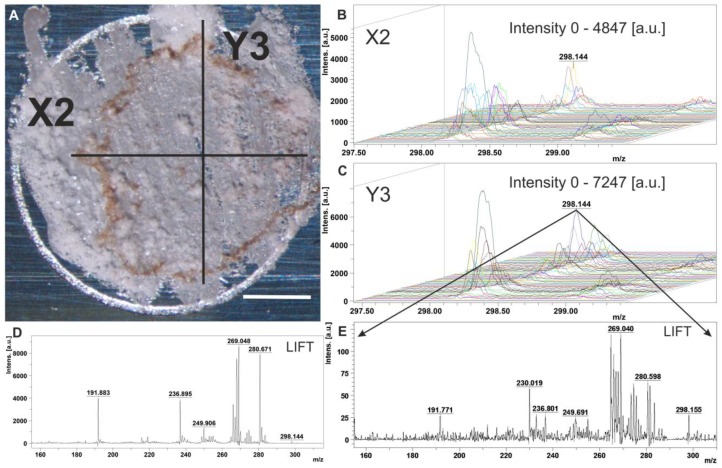
Direct matrix-assisted laser desorption/ionization mass spectrometric (MALDI-MS) analysis of a transverse section of the root nodule. (**A**) Image of the nodule with root sliced with raster (axis) direction. Bar, 10 mm. (**B**) MALDI-MS distribution of molecular mass *m*/*z* 298.142 across the nodule with root by raster X2. (**C**) MALDI-MS distribution of molecular mass *m*/*z* 298.142 across the root nodule by raster Y3. (**D**) MS/MS profile of precursor ions with *m*/*z* 289.144 from the root nodule. (**E**) MS/MS profile of precursor ions with *m⁄z* 289.144 from the standard solution. (a.u.), arbitrary units.

Direct MALDI-MS analysis was used to determine the spatial distribution of stepharine within the defined tissue structures. The longitudinal and transverse tissue slices from the different stages of the friable calli, SEs, and nodules with roots from the S2 and S3 lines were subjected to direct MALDI-MS analysis by lines (rasters). The MALDI-MS results showed the same distribution pattern of signal intensity from the extracted ions with *m*/*z* 289.144 through the slices of both cultures, which was confirmed by the HPLC data (Figure 4). The MS/MS measurements of selected precursor ions with *m*/*z* 289.144 based on the LIFT mode were also performed. A comparison between the achieved fragmentations in our samples, standard solution (Figure 5D,E), and the literature data showed similar patterns. Our experiments revealed various distribution patterns of stepharine along the line inside the differentiated structures (Figure 5A–C and Figure S2). The lowest levels of stepharine were observed in young undifferentiated calli and transverse slices of the middle portion of the embryo (S2). The abundance of ions *m*/*z* 289.144 was specific to the vascular tissue region of the developing roots and SEs (Figure S2C,D), whereas the core region and the epidermal tissue often contained minimal levels of the alkaloid, if at all. MALDI-MS-derived metabolite distributions were at their maxima in the upper portion of the nodules with root (up to 7500 a.u., Figure 5C), which contain cells rich in intracellular inclusions (Figure 3A).

### 2.5. MALDI-MS Analysis of Microdissected Tissue

To confirm these data and more precisely define the stepharine-accumulating cells, we isolated different cells and tissues using LCM. Based on the histological investigations, the following types of cells and tissues were analyzed: Epidermal and subepidermal cells, PC cells, PI cells, and VT cells. These tissues were easily distinguished from each other in transverse dry uncolored sections.

**Figure 6:**
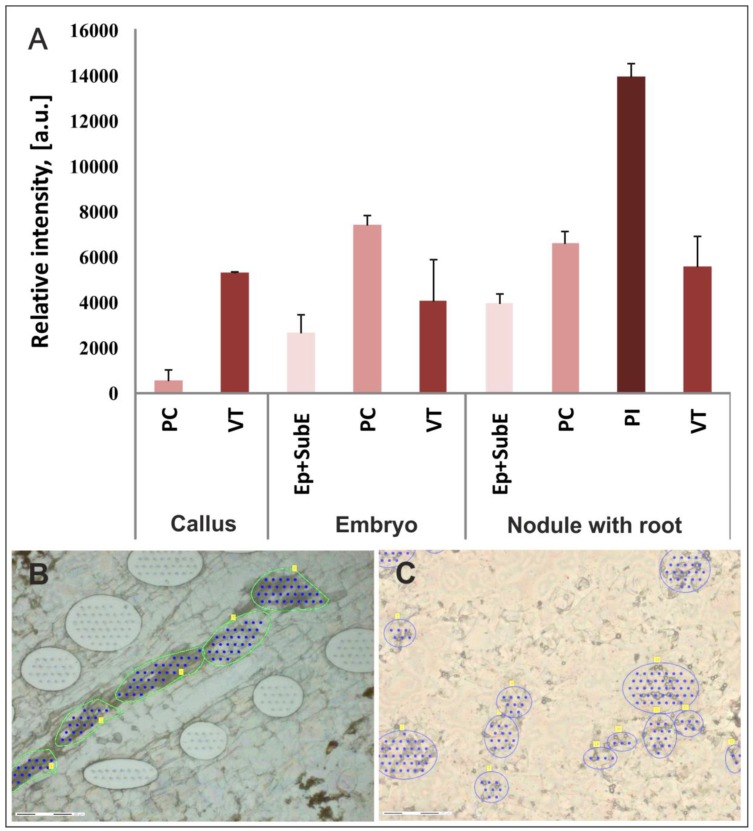
Matrix-assisted laser desorption ⁄ ionization mass spectrometric (MALDI-MS) data of stepharine (m⁄z 298.142) from microdissected tissue of the S2 line. (**A**) Diagram of stepharine content in the microdissected tissues. (**B**) Light microscopy image of microdissected portions of the vascular tissue with companion cells. (**C**) Light microscopy image of microdissected portions of the parenchymal cells with inclusions. PC, parenchymal cells; VT cells, vascular tissue with companion cells; PI cells, parenchymal cells with inclusions; Ep + SubE, epidermal and subepidermal tissue; (a.u.), arbitrary units. Blue and green lines indicate regions of extracted cells and blue dots marks the location of laser impulses. Bars, 150 µM.

The MALDI-MS spectra of extracts from LCM-dissected tissues showed stepharine at *m*/*z* 289.142 with the same distribution patterns as described for the MALDI MS experiments. In the microdissected samples, the main peak for stepharine was easily recognized and its content varied in different tissues and cells (Figure 6). The MS/MS spectra of protonated ions with *m*/*z* 289.142 from all samples showed fragment patterns typical of stepharine. The highest amount of stepharine was detected in the PI cells in the nodular portions of the roots. These tissues displayed a maximal intensity up to 14,000 units. The vascular tissues with companion cells from different structures had similar intensities. We concluded that the presence of PI cells is the main factor in the accumulation of this alkaloid.

## 3. Discussion

### 3.1. High Alkaloid Content as a Feature of Morphogenic Cell Cultures of S. Glabra

The engineering of plant cells with desirable alkaloid profiles in vitro typically leads to the perturbation of alkaloid metabolism and, thus, low yields. The S2 cell line described in this study is unique due to its high morphogenic activity. This line can be used for bioengineering purposes and as a model for systems biology. The content of stepharine in morphogenic tissues is very high and reaches 0.88–1.04% of the dry cell weight (Table 1). To our knowledge, this is the highest level of aporphine alkaloids obtained to date from plant cell cultures as well as from natural sources [35,36]. In addition to stepharine, the morphogenic cultures also contain ten other relevant alkaloids including magnoflorine, menisperine, roemerine, palmatine, corydalmine, N-methylcorydalmine, columbamine, tetrahydropalmatine, jatrorrhizine, and tetrandrine [18], although in much smaller quantities. How the cells sustain such a high load and its demand on the cell’s biosynthetic apparatus is not clear and represents an interesting phenomenon especially given there were no elicitors in the in vitro culture.

Furthermore, secondary somatic embryogenesis and rhizogenesis proceeded continuously. At the onset of SEs formation marked by the appearance of the procambial tissue and the first rudiments of the transport system (vascular elements), the synthesis of alkaloids begins; this was not observed in the meristematic cells of the parental S1 line (Figure 1). Apparently, alkaloids are transported throughout the body of the embryo, which is associated with the defensive role of alkaloids. Stepharine biosynthesis is likely to be constitutively activated at this stage of development. In this regard, we agree with the conclusions of Heinze et al. [1] that the advantages provided by alkaloids toward plant defense and communication must be balanced by effective management of the burdens inherent to secondary biosynthesis including competition for resources, metabolic derangement and potential self-intoxication. Self-intoxication is the most likely the reason of the failure of the induction of *S. glabra* calli on agarized nutrient media. Only specialized cells growing in liquid media that can tolerate the accumulation of alkaloids could circumvent this restriction.

### 3.2. Stepharine and Cell Differentiation: Matching Common Rules and Originality

According to our current understanding of alkaloid accumulation, most alkaloids are harvested in the laticifer system of plants’ aerial organs, which are tightly connected with vascular elements and their companion cells [14,22,37,38]. Some of the alkaloids accumulate constitutively in the roots with cell types other than laticifers [13,23,38]. Whether S. glabra possesses a laticifer system was unknown. In this study, we found single articulated anastomosing laticifer cells during the latest stages of somatic embryogenesis (Figure 4). According to the classification described by Hagel et al. [37], our type of alkaloid pattering most closely resembles the accumulation of protoberberine alkaloids in aerial organs of *Papaver somniferum* plants. However, unlike the Papaveraceae model, we observed the formation of laticifer cells and the appearance of PI cells only in hypocotyls (Figure 4). During SE development, the toxic load of alkaloids inside the cells increases. In this case, *S. glabra* plants, like many other plants that produce alkaloids, have adapted to the toxic pressure by forming an additional transport secretory (laticifer) system and depository cells (PI and VT cells) [14,22,37,38,39]. The most abundant accumulation of stepharine was observed in the hypocotyl portions of SEs and nodules with roots (Figure 4 and Figure 5). Such high stepharine levels in distinct tissue zones can be explained not only by local biosynthesis but also by transport from other cells. Because equal amounts of stepharine were found in SEs and roots at the later stages of development, we propose that stepharine accumulation depends on the quantity and proportion of the specific PI and VT cells (Figure S3). Thus, the primary reason for the high stepharine content in our morphogenic cell cultures is likely to be the presence of well-differentiated whole structures with hypocotyls regions. At this stage, the proportion of PI and VT cells in SEs and nodules with roots reached maximal levels.

The second reason is the formation of nodules. Histological investigation of the SEs showed that when the development of the apical meristem ceased, the nodules with main root provided sizeable deposit sites for stepharine (Figure 5E). In the hypocotyl portion of the main roots, vascular tissue swells and forms an abundance of PI cells (Figure 4E) with high stepharine content (Figure 5 and Figure 6). The main roots can produce secondary roots, but their development leads to decreasing amounts of stepharine because the secondary roots contain only VT cells and no additional hypocotyl portions with laticifers and PI cells (Figure 1B,C and Figure 3D).

### 3.3. Stepharine: Cell Culture vs. Whole Plants

In mature plants, a tuber develops from the hypocotyl region of embryos, whose cells represent the main depositories for stepharine and other alkaloids. Most likely, alkaloid transport is carried out along the horizontal axis of tubers as in the hypocotyl portions of SEs. There is no need to form depository cells in the differentiated organs of the plant as alkaloids are always in the transport system (i.e., VT cells) and, upon damage or injury, can be readily delivered from the PI cells of the tuber through the plant’s vasculature. The data published by Yi et al. [33] showed low levels of stepharine in the stem vascular tissue of *Sinomenium acutum*, which corroborates our results (Figure 1E,F). Micro-plants accumulated stepharine in their roots, stems and petioles in small amounts because they contain only VT cells. Developing organs do not accumulate a sufficient pool of alkaloids because the micro-plants have no depository sites. At all stages of development, stepharine accumulation is strictly organ-specific. Such regulation of alkaloid accumulation in plant cell cultures is reflective of the spatial-developmental regulation at the whole plant level. Indeed, it was shown previously that even if single highly specialized cells (laticifers) arise in cell cultures, these cultures are still not capable of accumulating high levels of alkaloids [24,25,26,27].

Castelblanque et al. [40] showed symmetric pairs of elongated laticifer initials in the early stages of laticifer cellular differentiation at the heart stage of embryo development and in the region from which future procambial tissue eventually differentiates. They observed that *Euphorbia lathyris* formed a well-developed laticifer system in close connection with the apical meristem to enable vertical alkaloid transport. Despite the fact that initials of laticifers and PI cells emerge at the same time and arise from the procambial portions of vascular tissues, deposited sites of stepharine are associated only with the root apex of SEs and the main roots. As such, *S. glabra* plants engage in horizontal stepharine transfer and then form a tuber. It is conceivable that different plants employ varied biosynthetic and accumulation strategies due to differential evolutionary pressures; this is in agreement with the theory of the polyphyletic origin of laticifers [14,39,40,41].

The signals that determine the spatial-developmental regulation of alkaloid biosynthesis are unknown. The main obstacle facing the field of alkaloid research in the coming years is the lack of major alkaloid groups in *Arabidopsis thaliana*. Research on non-model plants has unavoidable restrictions such as the inability to construct a network of protein-protein interactions to create a model for experimental verification. Nevertheless, much is already known about the transcriptional control of the biosynthetic genes for the main groups of alkaloids. Of note is the recent discovery of the ORCA transcription factors - members of the AP2/ERF transcription factor family [20,42] that are regulated by the basic helix–loop–helix transcription factor MYC2 [43]. MYC2 appears to play a central role in the interplay between secondary metabolism and hormone signaling [44]. These investigations hint at the involvement of the MYC2-JAZ-COI1 module in alkaloid biosynthesis in addition to regulatory factors identified in studies of polyphenols (e.g., MYC2-DELLA, -EIN3, -AHP5 interactions) as well as DELLA signaling which involved in procambium establishment [45].

## 4. Materials and Methods

### 4.1. Plant Material and Callus Cultures

Samples of *Stephania glabra* (ROXB.) Miers. (Menispermaceae) were collected in Experimental Field Station of Tay Nguyen Institute of Biology (Dalat, Vietnam). Callus and suspension cultures of *S. glabra* were established from young leaves of plants as described previously [18]. Five different cell lines were obtained (S1–S5).

Calli were cultivated in 100-mL Erlenmeyer flasks containing 30 mL of W_B/A_ medium [46] supplemented with the following components (mg L^−1^): thiamine-HCl (0.2), nicotinic acid (0.5), pyridoxine-HCl (0.5), meso-inositol (100), peptone (100), sucrose (25,000) and agar (6000). The calli were grown at 25 °C in the dark at 30-day subculture intervals. Suspension cultures were grown on a circular shaker in 0.5 l flasks containing 70–100 mL of cell suspension at 25 °C in the dark for 20 days. For light cultivation, we used luminescent white lamps with photosynthetic photon flux density at 49 µmol (m^2^s)^−1^ and 16 h: 8 h photoperiod.

The S1 and S2 lines were grown in liquid media with 2.4-dichlorophenoxyacetic acid (2,4-D, 0.5 mg L^−1^) (S1) or 6-benzyladenine (BA, 0.5 mg L^−1^) and α-naphthaleneacetic acid (NAA, 2.0 mg L^−1^) (S2). The S3, S4 and S5 lines were cultured on agarized media. The following combinations of growth regulators were used: 1.0 mg L^−1^ NAA (S3), 0.5 mg L^−1^ BA/1 mg L^−1^ NAA (S4), and 1.25 mg L^−1^ BA (S5).

All callus lines are maintained by subculturing in the Collection of Plant Cell Cultures at the Federal Scientific Center of the East Asia Terrestrial Biodiversity (Institute of Biology and Soil Science), Far Eastern Branch of the Russian Academy of Sciences (Vladivostok, Russia). The age of all cultures was 3 years.

### 4.2. Chemicals

Reagents for plant tissue culture were obtained from Sigma-Aldrich (St. Louis, MO, USA). Ethanol, methanol, *n*-hexane, water, formic acid, acetic acid, and acetonitrile (ACN) were of high-performance liquid chromatography (HPLC) grade and obtained from Merck (Darmstadt, Germany). Analytical standard of stepharine was isolated from callus cultures of *S. glabra* [17]. 2.5-dihydroxybenzoic acid (2.5-DHB) and trifluoroacetic acid (TFA) were purchased from Sigma-Aldrich (Stainheim, Germany).

### 4.3. Histological Analysis

After harvesting, samples from cell cultures were fixed immediately in FAA (3.7%: formaldehyde: 50% ethanol: 5% acetic acid) by vacuum infiltration, dehydrated, embedded in paraffin wax and sectioned at 8 µM thickness with a HM 340E rotary microtome (Microm, Thermo Scientific, Walldorf, UK). To correlate MALDI mass spectrometry with the histological analysis, the sections mounted onto microscopic slides were double-stained with hematoxilin and alcian blue and images were captured using AxioImager A1 microscope (Carl Zeiss, Jena, Germany).

### 4.4. Analytical Chromatography

Samples were prepared as described previously [17]. Briefly, after being dried to а constant weight and powdered, cell samples were extracted twice with a mixture of methanol: Water: Acetic acid (50:50:0.1, *v*:*v*:*v*) using an ultrasonic bath and shaking incubator. An Agilent Technologies 1260 Infinity Analytical HPLC system (Agilent Technologies, Santa Clara, CA, USA), equipped with a photodiode array detector and Zorbax C18 column (150 mm, 2.1-mm i.d., 3.5-μM portion size, Agilent Technologies), was used for HPLC analysis. The elution of alkaloids was carried out in gradient mode with water containing 15 mM sodium acetate and 35 mM acetic acid (Solvent A) and acetonitrile (Solvent B). The content of Solvent B was linearly changed as follows: 0 min 5%; 25 min 50%; 30 min 95%. The column was heated to a constant 40 °C, the flow rate was kept at 0.2 mL minute^−1^, and the separation was monitored at 237 nm for stepharine quantification. Liquid chromatography data were collected and processed using Agilent OpenLAB CDS software (v.01.06.111). To verify the accuracy of the determination of the target component, analyses were controlled using an ion trap mass spectrometer (Bruker HCT Ultra PTM Discovery System, Bruker Daltonik GmbH, Bremen, Germany) interfaced with an analytical HPLC system. The mass spectrometric (MS) data were recorded using electrospray ionization in the positive mode for ion detection. The settings were as follows: Flow rate of the drying gas (N_2_) 8.0 L minute^−1^, the nebulizer gas (N_2_) pressure 175 kPa, the ion source potential 4.0 kV, and the drying gas temperature 325 °C. MS/MS data were acquired in Auto-MS^2^ mode (smart fragmentation) by ramping the collision energy. The fragmentation amplitude was set to 1 V. The full scan ranges of *m*/*z* detection were 100–1000. MS data were collected using the Bruker Daltonics Compass 1.3 Esquire Control software (v.6.2.581.3) and processed with the Bruker Daltonics Compass 1.3 Data Analysis software (v.4.0.234.0).

### 4.5. Cryosectioning

To prepare tissues for cryostat sectioning, small pieces of calli and morphological structures were snap frozen in liquid nitrogen or frozen in a cryostat chamber at −25 °C. Prior to analysis, the tissue samples were equilibrated to −25 °C, followed by sectioning at −25 °C. Samples were sectioned at a thickness of 40–60 µm using Feather C35 80 mm blades (Feather Safety Razor, Osaka, Japan) in a Microm CryoStar NX 70 (Thermo Scientific, Loughborough, UK). Serial longitudinal and transversal sections were transferred directly onto an MTP 384 target plate ground steel (Bruker, Germany) with a 2.5-DHB matrix for MALDI-MS on SuperFrost glass microscope slides to monitor cell and tissue morphology and on PET membrane slides for microdissection (Thermo Scientific, Loughborough, UK). The slices were desiccated at room temperature for 45–60 min.

### 4.6. Laser-Capture Microdissection (LCM)

To isolate and harvest cells from tissue sections by laser microdissection, a PALM Laser Microbeam Instrument (Zeiss, Bernried, Germany) was employed. The operation of the settings for laser focus, laser power, and Laser Pressure Catapulting (LPC) functions was achieved through the PalmRobo 4.5 software. The cell material was separated from three cryosections for each of the three biological replicates. Undifferentiated calli, epidermal and subepidermal tissues, vascular tissue with companion cells (VT cells), normal parenchymal cells (PC cells) and parenchymal cells with inclusions (PI cells) were collected into 0.2 µL microtubes with adhesive lids (Zeiss, Bernried, Germany). The area of the microdissected materials for each sample was around 500,000 µM^2^. Collected samples were used immediately or stored at −20 °C until processing.

### 4.7. Matrix Deposition

A matrix solution was chosen based on Peukert et al. [47] with fewer noises for optimal reproducibility. 2.5-DHB at a concentration of 7 mg mL^−1^ in 1:1 (*v*:*v*) ACN: 0.2% TFA was manually pipetted onto the target plate and left to form a thin layer in open air at room temperature for 10 min. To prevent re-localization of stepharine, the slices were transferred onto the target plate after drying the matrix, and then the plate with the slices was put into a desiccator to dry for 30 min and analyzed immediately. To minimize degradation, 4 µL of the microdissected material in the 2.5-DHB matrix was directly re-suspended on the adhesive lid and immediately deposited onto the target plate and dried.

### 4.8. MALDI-MS Data Acquisition and Analysis

An Autoflex^TM^ speed MALDI-TOF with a nitrogen laser operated in the positive reflector mode (standard method RP_Imaging_200-1500_Da.par) (Bruker Daltonics, Bremen, Germany), in the range of 0–500 Da, was used for MS acquisitions. Instrument parameters were set using the FlexControl software (version 3.4, Bruker Daltonics, Bremen, Germany). Mass calibration was performed externally using a stepharine standard. The purified standard of stepharine was deposited on a MALDI target to determine optimum MS parameters. For direct tissue assessment of stepharine, slices of different callus structures from the target plate were analyzed with the help of a raster array in both the x and y dimensions with a spatial resolution of approximately 40 µM created by the FlexControl software (version 3.4, Bruker Daltonics, Bremen, Germany) (Figure S1). Each representative mass spectrum of stepharine was averaged from 300 laser shots. Peaks were labeled and processed using PMF.FAMSMethods at FlexAnalysis 3.4 software (Bruker Daltonics, Bremen, Germany). The mass profiles were recorded by MALDI-MS using the same acquisition parameters for both the tissue slices and the microdissected material. For MS/MS analyses, we used the SNAP_full_process.FALIFTMethod. Each MS/MS spectrum was obtained by averaging 1500–3000 laser shots (300 shots per step) acquired at minimum laser power and sequence interpretation was performed manually.

### 4.9. Statistical Analysis

Average values and standard errors were calculated using the STATISTICA 9 program.

## 5. Conclusions

In summary, our study shows that morphogenic cell cultures of *S. glabra* not only effectively produce stepharine in great amounts, but also allow the obtaining of a large number of micro-propagated plants. Research at the cellular level using mass spectrometry allows a better understanding of the distribution of alkaloids and may help in the development of new plant cell cultures for stable production of aporphine alkaloids. Further research should be directed to the study of the relationship between signaling systems that regulate alkaloid biosynthesis and those that regulate cellular differentiation, growth, and development.

## Figures and Tables

**Table 1:** The phenotype and the main biotechnological parameters of the *S. glabra* lines (dark conditions) and rooted plants.

Cell Line	Phenotype	Fresh Biomass (g L^−1^)	Dry Biomass (g L^−1^)	Stepharine Content (%DW)	Stepharine Production (mg L^−1^)
S1 Liquid Homogenous	Undifferentiated cell aggregates	161.6 ± 24.2	11.8 ± 1.4	-	-
S2 Liquid Heterogeneous	Somatic embryos	453.3 ± 31	16.8 ± 0.6	0.88 ± 0.06	147.0 ± 12.6
	Nodules with roots			1.04 ± 0.03	175.3 ± 5.7
S3 Solid Heterogeneous	Somatic embryos	78.75 ± 8.06	4.6 ± 0.49	0.33 ± 0.01	15.3 ± 3.2
	Nodules with roots			0.80 ± 0.19	36.9 ± 8.7
S4 Solid Heterogeneous	Plantlet leafs	119.8 ± 8.3	6.7 ± 0.4	-	-
	Petiole			0.23 ± 0.04	115.4 ± 2.7
	Main stems			0.29 ± 0.09	19.1 ± 6.4
	Plantlets roots			0.66 ± 0.06	44.2 ± 4.0
S5 Solid Heterogeneous	Deformed Stems	102 ± 15.2	7.7 ± 0.76	0.20 ± 0.01	15.7 ± 0.7

Suspension culture S1 was cultivated for 10 days, S2 for 25 days and the callus culture S3, S4, S5 for 30 days. The data (mean ± SE) was obtained from six independent experiments with 10 replicates each.

## Supplementary Materials

Supplementary materials can be found at http://www.mdpi.com/1422-0067/20/4/808/s1. Figure S1. Raster array in both x and y dimensions on an MTP 384 target plate, Figure S2. Raster matrix-assisted laser desorption ⁄ionization mass spectrometric (MALDI-MS) analysis of differentiated structures in the S2 line, Figure S3. Scheme of stepharine deposit site formation in vivo and in vitro (yellow spots – initials of laticifers and PI cells; red lines – deposit sites of stepharine (PI cells).
