# Supplementary material for: Tempo-Spatial Pattern of Stepharine Accumulation in Stephania Glabra Morphogenic Tissues
Source: Int J Mol Sci. 2019 Feb 13;20(4):808. doi: 10.3390/ijms20040808 (PMC6413192; doi:10.3390/ijms20040808)
Supplement: Supplementary file 1 [file ijms-20-00808-s001.pdf]

Supplementary materials

Tempo-spatial pattern of stepharine accumulation in *Stephania glabra* morphogenic tissues

Tatiana Y. Gorpenchenko, Valeria P. Grigorchuk, Dmitry V. Bulgakov, Galina K. Tchernoded, Victor P. Bulgakov

Supplementary Figures

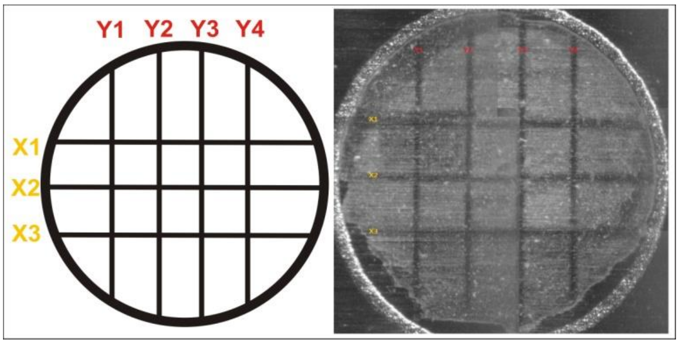

Figure S1. Raster array in both x and y dimensions on an MTP 384 target plate. Ground steel well. Standard diameter 40 mm.

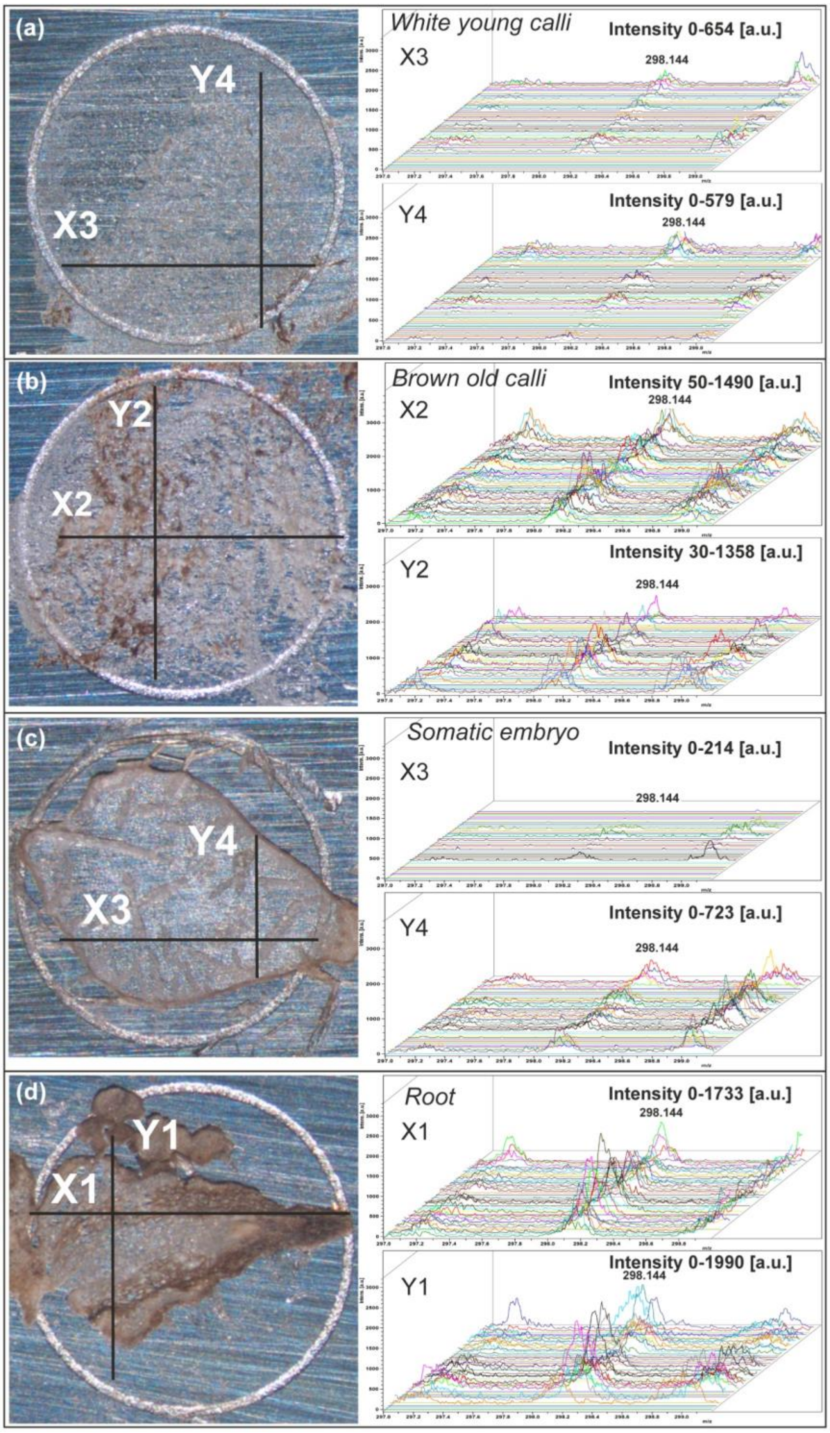

Figure S2. Raster matrix-assisted laser desorption ⁄ionization mass spectrometric (MALDI-MS) analysis of differentiated structures in the S2 line. (a) Image of the young calli and MALDI-MS distribution of the molecular mass *m/z* 298.142 across young calli by raster X3, Y4. (b) Image of the mature calli and MALDI-MS distribution of the molecular mass *m/z* 298.142 across this tissue by raster X2, Y2. (c) Image of the somatic embryo and MALDI-MS distribution of the molecular mass *m/z* 298.142 across this structure by raster X3, Y4. (d) Image of the main root and MALDI-MS distribution of molecular mass *m/z* 298.142 across this tissue by raster X1, Y1. [a.u.], arbitrary units.

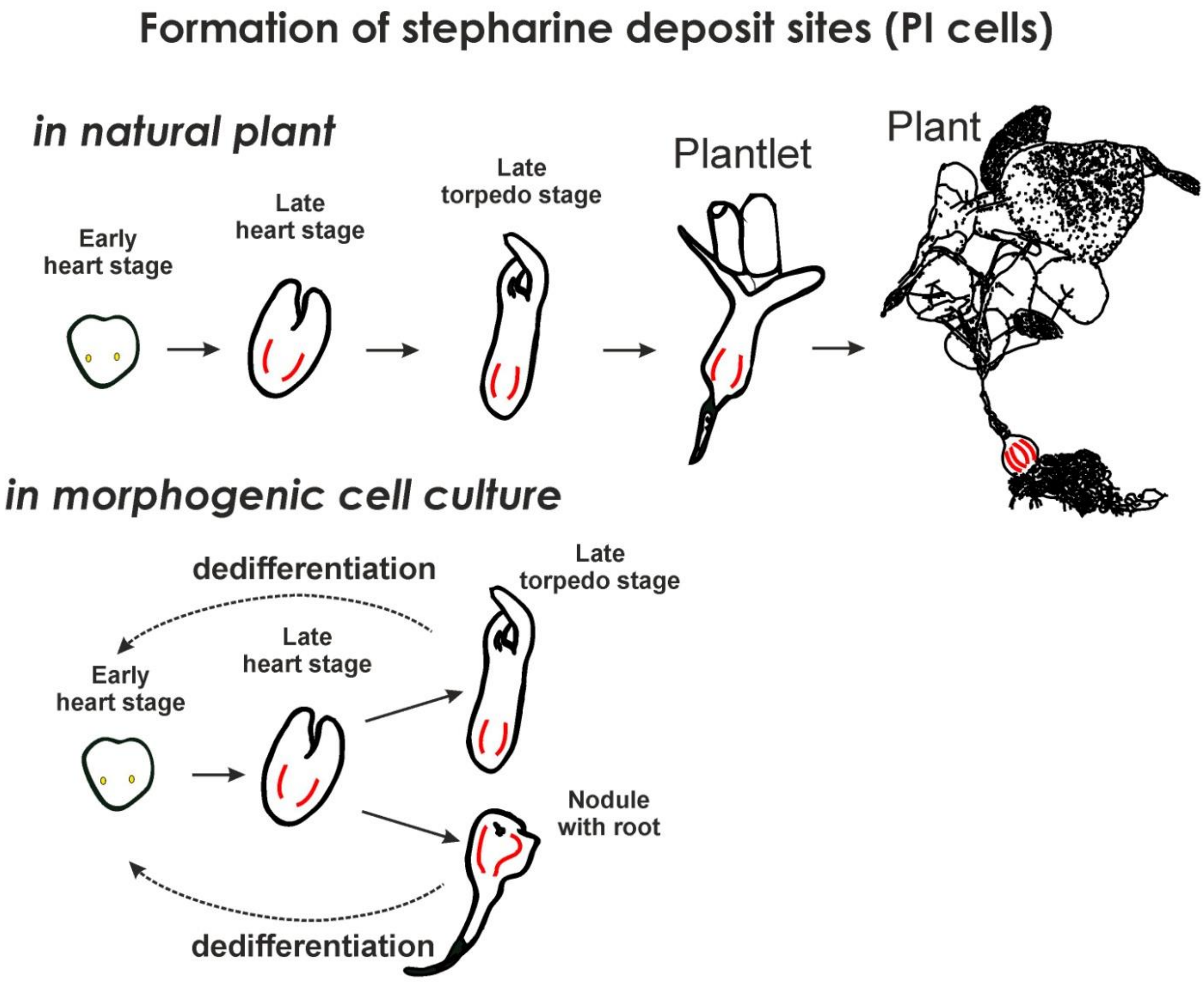

Figure S3. Scheme of stepharine deposit site formation *in vivo* and *in vitro* (yellow spots – initials of laticifers and PI cells; red lines – deposit sites of stepharine (PI cells). .
